# Pleomorphic adenoma of the lip: a case report and literature review

**DOI:** 10.1093/jscr/rjaf311

**Published:** 2025-05-15

**Authors:** Rawa M Ali, Rebaz M Ali, Aras J Qaradakhy, Dilan S Hiwa, Ari M Abdullah, Diyar A Omar, Shko H Hassan, Abdullah A Qadir, Abdulwahid M Salih, Fahmi H Kakamad

**Affiliations:** Scientific Affairs Department, Smart Health Tower, Madam Mitterrand Street, Sulaymaniyah 46001, Iraq; Hospital for Treatment of Victims of Chemical Weapons, Mawlawy Street, Halabja 46018, Iraq; Scientific Affairs Department, Smart Health Tower, Madam Mitterrand Street, Sulaymaniyah 46001, Iraq; Scientific Affairs Department, Smart Health Tower, Madam Mitterrand Street, Sulaymaniyah 46001, Iraq; Department of Radiology, Shorsh Teaching Hospital, Shorsh Street, Sulaymaniyah 46001, Iraq; Scientific Affairs Department, Smart Health Tower, Madam Mitterrand Street, Sulaymaniyah 46001, Iraq; Scientific Affairs Department, Smart Health Tower, Madam Mitterrand Street, Sulaymaniyah 46001, Iraq; Department of Pathology, Sulaymaniyah Teaching Hospital, Zanko Street, Sulaymaniyah 46001, Iraq; Medical Laboratory Technology, Shaqlawa Technical College, 120 Meter Street, Erbil Polytechnic University, Erbil 44001, Iraq; Kscien Organization for Scientific Research (Middle East Office), Hamdi Street, Azadi Mall, Sulaymaniyah 46001, Iraq; Scientific Affairs Department, Smart Health Tower, Madam Mitterrand Street, Sulaymaniyah 46001, Iraq; Scientific Affairs Department, Smart Health Tower, Madam Mitterrand Street, Sulaymaniyah 46001, Iraq; Scientific Affairs Department, Smart Health Tower, Madam Mitterrand Street, Sulaymaniyah 46001, Iraq; College of Medicine, University of Sulaimani, Madam Mitterrand Street, Sulaymaniyah 46001, Iraq; Scientific Affairs Department, Smart Health Tower, Madam Mitterrand Street, Sulaymaniyah 46001, Iraq; Kscien Organization for Scientific Research (Middle East Office), Hamdi Street, Azadi Mall, Sulaymaniyah 46001, Iraq; College of Medicine, University of Sulaimani, Madam Mitterrand Street, Sulaymaniyah 46001, Iraq

**Keywords:** benign mixed tumor, neoplasm, salivary gland, lip tumor

## Abstract

Pleomorphic adenoma (PA) most commonly affects the major salivary glands but can also involve the minor salivary glands, particularly those of the hard and soft palate. However, the current study aims to present a case of upper lip PA. A 29-year-old male presented with a painless, firm, slow-growing swelling (1.5 × 1.5 cm) on the left upper lip. Ultrasonography showed a solid, hypoechoic nodule with irregular margins and mild peripheral vascularity. The encapsulated tumor was excised, and histopathology confirmed PA. At the 7-month follow-up, no evidence of recurrence was observed. Though typically seen in adults aged 40–70, PA can affect younger patients. Complete excision with clear margins is essential due to the risk of recurrence or malignant transformation. PA should be considered in the differential diagnosis of upper lip swellings.

## Introduction

Salivary glands are divided into major (parotid, sublingual, submandibular) and minor glands, with the latter located in areas like the oral cavity, nasal passages, and oropharynx [1, 2]. Salivary gland tumors represent 3% of head and neck tumors, with pleomorphic adenoma (PA) being the most common benign neoplasm [1, 3]. Minor salivary gland PAs typically affect the soft and hard palates, reflecting the higher embryological distribution of minor salivary glands in these areas compared to the lips, making upper lip involvement a rare occurrence [2, 4–6]. This study reports a case of a 29-year-old male with upper lip PA. Only peer-reviewed papers were included, and the study was written according to CaReL guidelines [7, 8].

## Case presentation

### Patient information

A 29-year-old male presented with a left anterior upper lip swelling. He had right-sided weakness and spasticity, along with intellectual disability, attributed to an ischemic insult sustained during birth. Family and surgical histories were negative.

### Clinical finding

**Figure 1:**
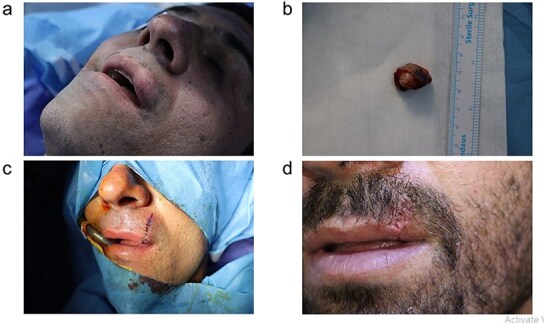
(a) Preoperative appearance of the mass located in the upper lip. (b) The excised mass. (c, d) The postoperative appearance of the patient.

On physical examination, the mass measured approximately 1.5 × 1.5 cm, and it was well-defined, sessile, and firm. The mass had expanded outwards towards the skin rather than inwards towards the oral mucosa. The skin over the mass appeared stretched and had turned pink. No noticeable changes were present in the surrounding area, and there was no regional lymph node enlargement (Fig. 1). The patient also had multiple skin lesions and skin tags.

### Diagnostic approach

Ultrasonography revealed a solid, hypoechoic nodule in the left upper lip, measuring 12 × 10 × 9 mm, with irregular margins, mild peripheral vascularity (having a high resistive index of ~1), and full-thickness involvement, features that were highly suspicious. A biopsy was taken, and the histologic findings were suggestive of a pleomorphic adenoma.

### Therapeutic intervention

**Figure 2:**
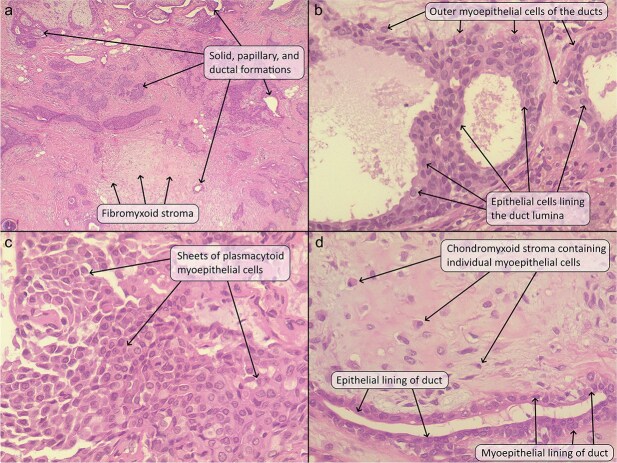
(a) The tumor comprised of solid clusters and ductal structures (some of which showed intraluminal papillary formations) lying within a fibrous and chondromyxoid stroma. (b) The ductal structures were lined by a luminal layer of epithelial cells and an abluminal layer of myoepithelial cells. (c) Some areas showed sheets of plasmacytoid myoepithelial cells with bland nuclei. (d) The chondromyxoid stroma contained individual plasmacytoid myoepithelial cells and ductal structures. Hematoxylin and eosin stain; original magnification 40× (a), 400× (b–d).

Under general anesthesia and through a longitudinal incision, the fully encapsulated tumor was wholly excised with ease. A layered repair was carried out, involving the closure of the deep dermal and epidermal layers to ensure proper alignment and achieve satisfactory cosmetic outcomes. Gross examination showed a well-defined, white nodule near the vermilion border measuring 1.9 cm in greatest dimension (Fig. 1). Histopathologic examination showed a relatively well-defined nodule under the skin that was composed of variably sized ductal formations showing cystic change, papillary growths, and solid formations, composed of cells that had a small amount of lightly eosinophilic cytoplasm and oval nuclei with fine chromatin and inconspicuous nucleoli. Some of the ducts showed squamous metaplasia, and some contained an eosinophilic secretion. These formations lay within a fibrous and chondromyxoid stroma containing clusters and individual cells with a plasmacytoid, spindled, and stellate configuration, having oval nuclei and fine chromatin. There was a giant cell reaction around the tumor. There was no significant mitotic activity, pleomorphism, or necrosis (Fig. 2).

### Follow-up and outcome

The patient had no intraoperative complications and had an uneventful postoperative period. At the 7-month follow-up, no evidence of recurrence was observed.

## Discussion

The parotid gland is the most common site for PA, comprising 80% of cases, followed by the submandibular gland (10%) [1]. PA can also arise in minor salivary glands, including the palate, buccal mucosa, nasal cavity, and tongue, but lip involvement is rare. The upper lip is six times more frequently affected than the lower lip, and it has been postulated that the disparity in the incidence of PA between the upper and lower lips can be attributed to embryological development. Specifically, the upper lip arises from the fusion of three facial protuberances, while the lower lip forms from the fusion of only two. This more complex fusion process in the upper lip increases the likelihood of ectopic embryonic cells becoming entrapped, thereby raising the risk of tumorigenesis in that region. Furthermore, the upper lip contains a greater number of well-developed labial salivary glands, in contrast to the lower lip, which harbors only a limited number of smaller glands. This anatomical difference may further account for the higher prevalence of PA in the upper lip [4, 9]. While PA’s pathogenesis remains unclear, the presence of chromosomal translocations with breakpoints at 8q12 and 12q13-15 is well recognized. These rearrangements lead to gene fusions involving key transcription factor genes, most notably PLAG1 and HMGA2. It has been revealed that HMGA2 acts as an upstream regulator of PLAG1, and that it modulates IGF2 expression through this transcription factor. This discovery offers a novel mechanistic insight into the aforementioned chromosomal abnormalities observed in PA, highlighting IGF2 as a key oncogenic driver and a potential therapeutic target [10]. Although Chiboub *et al*. reported a female predominance, recent studies show male cases are also frequent, with no clear sex predilection established (Table 1) [1, 2, 4, 11–14].

**Table 1:** Characteristics of patients with upper lip pleomorphic adenoma

**Author/year of publication**	**No. of cases**	**Country**	**Age (years)**	**Sex**	**Significant past history**	**Presentation**	**Side**	**Size (mm)**	**Histopathology**	**Management**	**Follow-up (month)**	**Recurrence**
Mamat *et al*./2020 [11]	1	Malaysia	4	Male		Painless lump for 3 years.	Central	15 × 10	Partially encapsulated pleomorphic adenoma with variable epithelial and stromal components.	Surgical excision	6	No
Kazikdas *et al*./2020 [1]	1	Cyprus	20	Male		Gradually enlarging painless mass for 2 years.	Right	40 × 30	Well-circumscribed submucosal lobular tumor consisting of ductal tubules lined by cuboidal cells, with adjacent areas of chondroid and myxoid stroma and proliferation of clusters of epithelial cells.	Surgical excision with abdominal fat harvested through a 15-mm inferior umbilical incision and inserted into the excision site as a volume filler.	12	No
Gelidan *et al*./2021 [3]	1	Saudi Arabia	46	Male	No	2-year history of painless, progressive upper lip external swelling.	Left	15 × 10	Circumscribed benign minor salivary gland neoplasm that shows epithelial (ductal) component forming the inner layer of cyst and tubules and myoepithelial cells present as the outer layer of cysts and tubules and scattered within the myxoid stroma.	Surgical excision	12	No
Suka *et al*./2021 [12]	1	Japan	54	Female	No	Painless slow-growing mass for 5 years.	Right	10 × 10	The tumor tissue consisted of densely proliferating stromal and epithelial cells forming ductal structures. The stroma exhibited myxomatous and hyalinizing degeneration, with some fibrous tissues present, but no cartilaginous tissue was observed.	Surgical excision	24	No
Qureshi *et al*./2022 [13]	1	Pakistan	39	Male	No	Painless gradually progressive swelling for 2 years.	Right	15	Circumscribed, encapsulated, biphasic neoplasm composed of stromal and epithelial components.	Surgical excision		
Chiboub *et al*./2023 [14]	1	Tunisia	24	Male	Thyroglossal duct cyst	Painless swelling on the lip for 5 years duration.	Left		Polygonal and spindle-shaped myoepithelial cells in a variable background stroma containing mucoid and myxoid areas.	Surgical excision	28	No
Alrehaili *et al*./2023 [4]	1	Saudi Arabia	24	Female	No	Painless lump and progressive swelling of the lip for 18 months.	Right	5 × 4 × 3	An island and sheets of plasmacytoid cells arranged in duct-like structures, with irregular tubules and patterned strands of benign neoplastic cells surrounded by chondromyxoid stroma. Some cystic areas are identified, with variable stroma exhibiting fibrous, chondroid, and myxoid appearance.	Surgical excision	12	No

PA typically presents in individuals aged 40–70, but cases in children, such as a 4-year-old male reported by Mamat *et al*., highlight its occurrence across all ages [11, 14]. Though histologically benign, PA can mimic malignancy due to features like capsule invasion, necrosis, increased cellularity, and cytologic atypia, complicating diagnosis [4]. Its slow-growing, painless nature often leads to delayed presentation, with patients typically seeking medical care more than two years after first noticing the mass. This underscores the need to consider PA in the differential diagnosis of lip swellings in both pediatric and adult patients [4, 11, 14].

As PA grows, surgery becomes more challenging, and recurrence risk rises. Surgical excision with clear margins is the standard treatment. Kazikdas *et al*. used abdominal fat grafting to restore lip contour postexcision [1, 4, 14]. Malignant transformation occurs in 2%–23% of cases, requiring wider margins (1–2 cm) when suspected [1, 4]. Despite being benign, PA demands close follow-up due to the risk of recurrence from capsular rupture during surgery [14]. Although no recurrences were noted in the reviewed cases with one-year follow-up, longer monitoring is necessary to rule out late recurrence. In conclusion, this rare presentation highlights that the differential diagnosis for upper lip swellings should include PA. Complete surgical excision may result in a good outcome, but close follow-up is recommended to monitor for any signs of recurrence.
